# Sudden, unexpected death due to glioblastoma: report of three fatal cases and review of the literature

**DOI:** 10.1186/1746-1596-8-73

**Published:** 2013-05-02

**Authors:** Irene Riezzo, Rosanna Zamparese, Margherita Neri, Francesco De Stefano, Ruggero Parente, Cristoforo Pomara, Emanuela Turillazzi, Francesco Ventura, Vittorio Fineschi

**Affiliations:** 1 Department of Forensic Pathology, University of Foggia, Ospedale “C. D’Avanzo”, viale degli Aviatori, 1, Foggia 71100, Italy; 2 Department of Legal Medicine, University of Genova, via de’ Toni 12, Genova 16132, Italy

**Keywords:** Glioblastoma, Sudden death, Immunohistochemistry stains, Western blotting

## Abstract

**Abstract:**

Sudden death from an undiagnosed primary intracranial neoplasm is an exceptionally rare event, with reported frequencies in the range of 0.02% to 2.1% in medico-legal autopsy series and only 12% of all cases of sudden, unexpected death due to primary intracranial tumors are due to glioblastomas. We present three cases of sudden, unexpected death due to glioblastoma, with different brain localization and expression. A complete methodological forensic approach by means of autopsy, histological and immunohistochemical examinations let us to conclude for an acute central dysregulation caused by glioblastoma and relative complication with rapid increase of intracranial pressure as cause of death. Although modern diagnostic imaging techniques have revolutionized the diagnosis of brain tumors, the autopsy and the careful gross examination and section of the fixed brain (with coronal section) is still the final word in determining exact location, topography, mass effects and histology and secondary damage of brain tumor and contributed the elucidation of the cause of death. Immunohistochemistry and proteomic analysis are mandatory in such cases.

**Virtual slides:**

The virtual slide(s) for this article can be found here: http://www.diagnosticpathology.diagnomx.eu/vs/1218574899466985

## Background

Glioblastoma is the most common malignant primary brain neoplasm, representing about 12-20% of all intracranial tumors and accounting for about 50-60% of all astrocytic neoplasms [1]. The astrocytic neoplasms occur in patients of all ages and arise at all levels of the neuraxis. In adults, most occur in the cerebral hemispheres, whereas in children typically occur in the brain stem [2-5] or thalamus [6-9]. Less commonly affected sites in both children and adults include the spinal cord [10-13] and cerebellum [14-16]. The 2007 World Health Organization (WHO) grading system designed three lesions of diffusely infiltrating astrocytic tumors: diffuse astrocytoma (grade II), anaplastic astrocytoma (grade III) and glioblastoma (grade IV) [17]. Among diffusely infiltrative astrocytomas of the cerebral hemispheres, a close correlation is observed between histologic grade and clinical variables: patient age, duration of symptoms and neurologic performance status. With occasional exceptions, lesions in older patients are more anaplastic, biologically aggressive, recently symptomatic, and destructive of neurologic function.

Sudden death from an undiagnosed primary intracranial neoplasm is an exceptionally rare event, with reported frequencies in the range of 0.02% to 2.1% in medico-legal autopsy series [18-24] and only 12% of all cases of sudden unexpected death due to primary intracranial tumors are due to glioblastomas [25].

We report three cases of sudden unexpected death due to undiagnosed glioblastoma grade IV according to WHO [17] with different brain localization and expression. Complete histological, immunohistochemical and proteomic examinations are presented, to improve diagnosis.

## Case presentation

### First case

**Figure 1:**
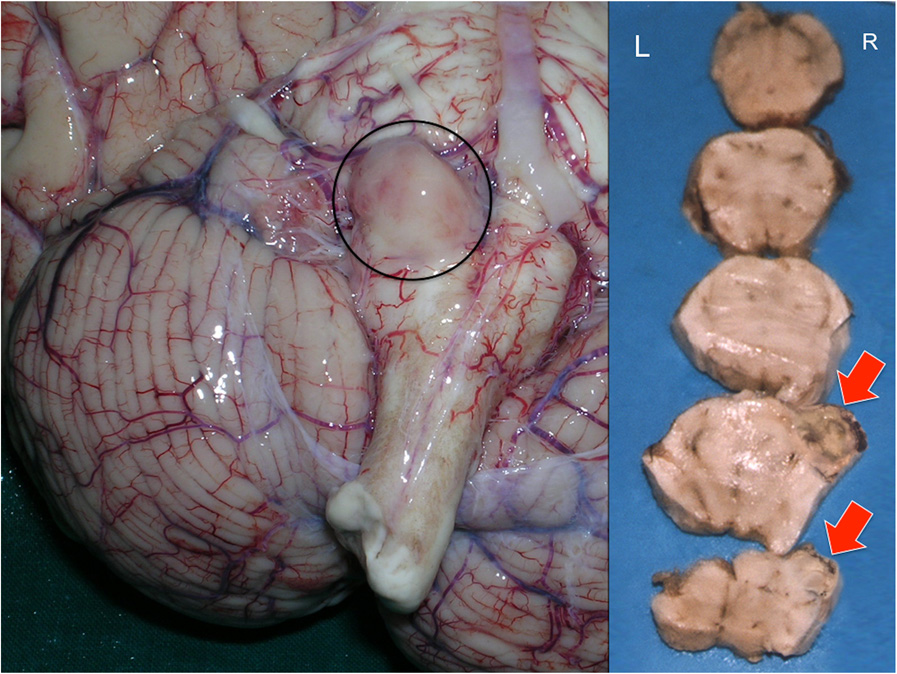
**Case 1: the brain weighed 1550 g and measured cm 19×16.5×6 and showed stasis and massive edema.** A spherical gelatinous solid mass (black circle), measuring 1 cm in diameter was attached in the right brain stem.

A 71-year-old-Caucasian man, with a past history of hyposthenia of the right arm, cervical spine surgery, chronic kidney disease and hepatic steatosis, showed headache, confusion state and difficulty in walking therefore he was transferred to the local hospital. The neurological examination revealed poor general condition, marked weight loss, ataxia and slowdown ideomotor, apathy, fatigue, lack of initiative. The laboratory examination of blood and liquor was negative for infection/inflammatory disease. To diagnose a multi**-**infarct dementia the patient was scheduled for TC and magnetic resonance imaging of the brain and the entire spine, but suddenly died prior to the imaging examination. At autopsy, moderate pulmonary edema and polyvisceral stasis were observed. The brain weighed 1550 g and showed massive edema. A spherical gelatinous solid mass, measuring 1 cm in diameter was attached in the right medulla (Figure 1). On coronal sections, the right temporal lobe showed a reddish-rusty mass lesion, measuring 1×2 cm and the third ventricle was compressed and dislocated. A small fragment of the mass was frozen for Western blot.

### Second case

**Figure 2:**
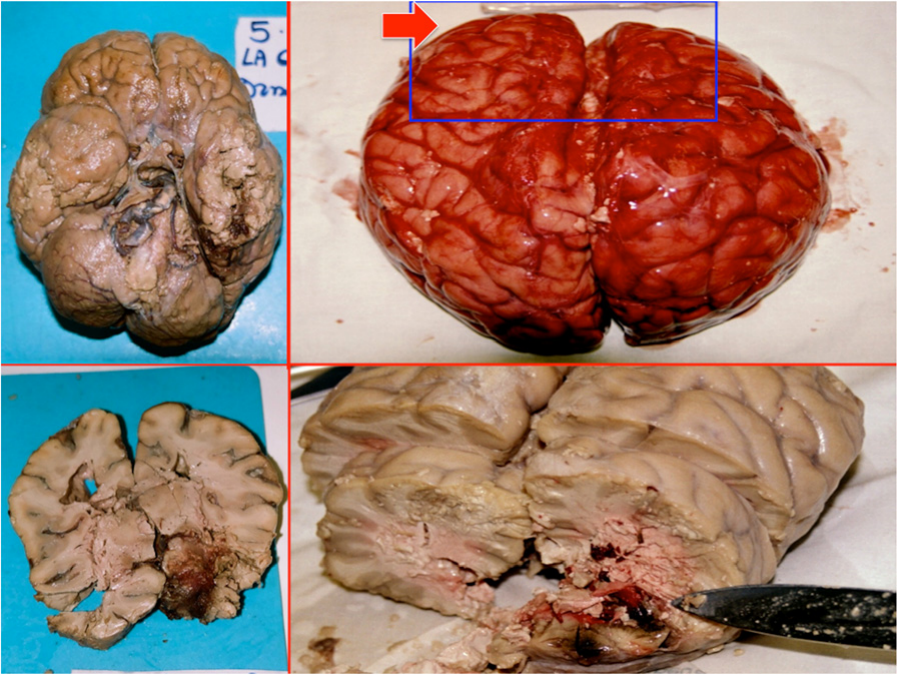
Case 2: on coronal section, the left temporal lobe showed a large necrotic hemorrhagic mass lesion, which measured cm 3×2.5×2.2, surrounded by edematous tissue.

A 79-year-old Caucasian man, with a history of ischemic heart disease and hypertension, was brought to the Hospital in the neurological unit for symptoms such as confusion, slackening, sleepiness, and tremor of the upper limbs started few days before. The brain CT scanner examination shows a large hypodense mass in the left temporal lobe with massive oedema and compression phenomena on occipital and temporal lobe and midline shift. The patient was then referred for neurosurgical consultation, but the day before surgery he suddenly died. General autopsy performed 48hs after death was unremarkable. The brain weighed 1600 g and measured cm 22×16×6.5, showed diffusely swollen cerebral hemispheres, an increase in volume of the left temporal lobe (Figure 2). There was no herniation of the temporal lobe, unci or cerebellar tonsils. On coronal section, after fixation, the left temporal lobe showed a large mass lesion, which measured cm 3×2.5×2.2, hemorrhagic and surrounded by necrotic and oedematous tissue.

### Third case

A 43-year-old Polish man was found dead in a slope near the track of the railway. Death scene investigation was unremarkable. A complete autopsy was performed 48 hs after death. The external examination revealed only same abrasions and bruises on the face, and the upper and lower limbs. The internal examination revealed polyvisceral stasis, heavy lungs and reddish colored foam on trachea and the main bronchi. The skull was entire. The examination of the brain (cm 21×16×6, g 1630) after fixation in buffered formalin revealed a cerebral edema and an increase in volume of the left frontal lobe. On coronal sections, the cerebral hemispheres were asymmetrical with deviation of midline structures from left toward right. In the left frontal lobe a spherical mass (cm 3.5×3×1.5), with variegated appearance and contained regions of necrosis and haemorrhage was found. The blood alcohol concentration was 0.8 g/l.

#### Histology

**Figure 3:**
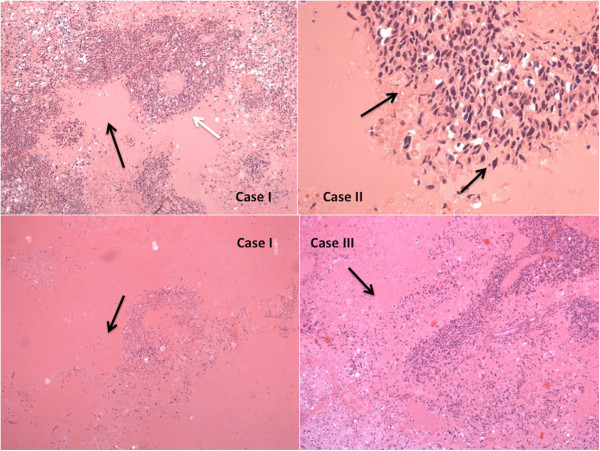
Samples taken from right brain stem and temporal lobe (case I), left temporal lobe (case II), and from left frontal lobe (case III): we observed dense cellularity, shrinking pleomorphism, and zones of coagulative necrosis lined (white arrow) by “palisading” tumor cells with nuclear pleomorphism.

**Figure 4:**
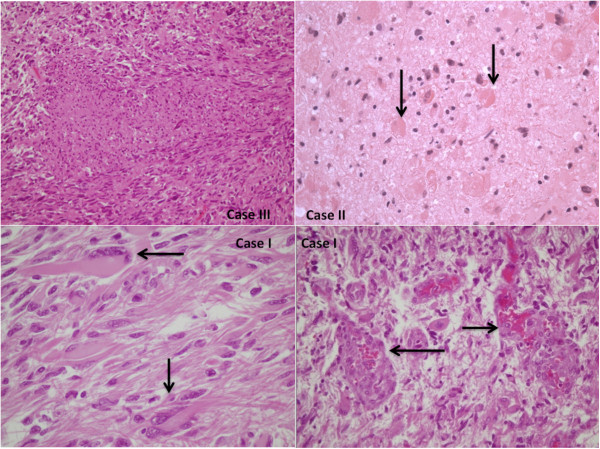
Cells (arrows) within deeply eosinophilic masses (cases 2 and 3), complex, “glomeruloid” quality of the microvascular proliferation (arrows) (proliferating blood vessels come to be lined by cells heaped up in disorderly fashion and are transformed into glomeruloid or solid tufts), multinucleated giant cells (case 1).

In all cases, the etiopathogenetic definition was outlined by histological examinations performed on brain tissue samples using haematoxylin-eosin (H&E) and Perl’s and revealed the presence of diffuse and marked cytotoxic and vasogenic brain edema, and in samples taken from right medulla and temporal lobe (case I), left temporal lobe (case II), left frontal lobe (case III) foci of central necrosis surrounded by neoplastic cells with nuclear pleomorphism, pseudopalisading, multinucleated cells (“giant cells glioblastomas”) and vascular proliferation (Figures 3, 4). Areas of extensive haemorrhage near tumour cells were also observed.

#### Immunohistochemistry

**Figure 5:**
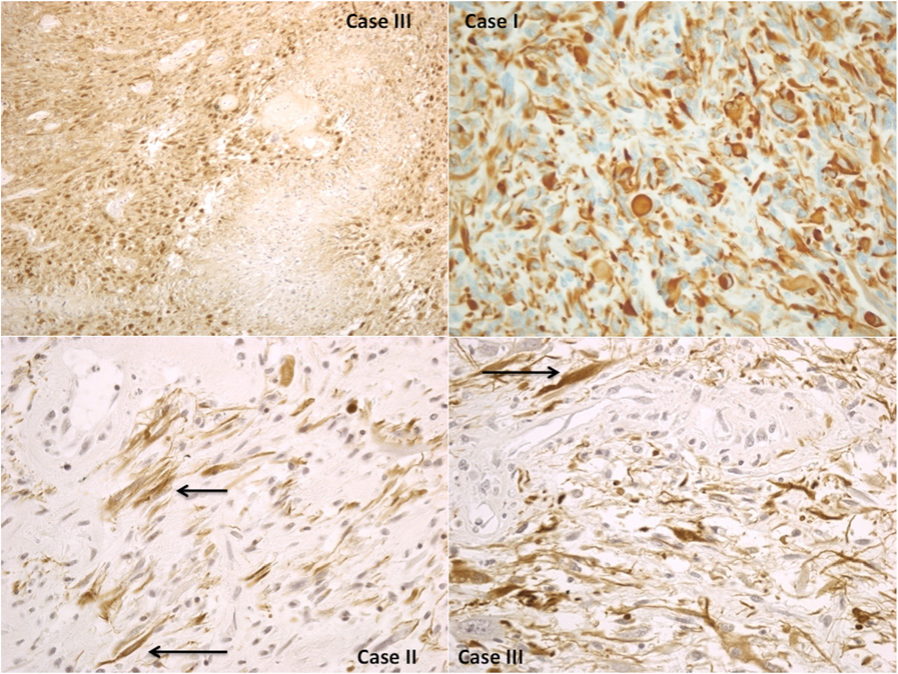
**The immunohistochemical examination of the brain specimens revealed a positive reaction for antibodies anti-GFAP (glial fibrillary acidic protein).** Small elongated cells and extreme cytologic pleomorphism were evident.

**Figure 6:**
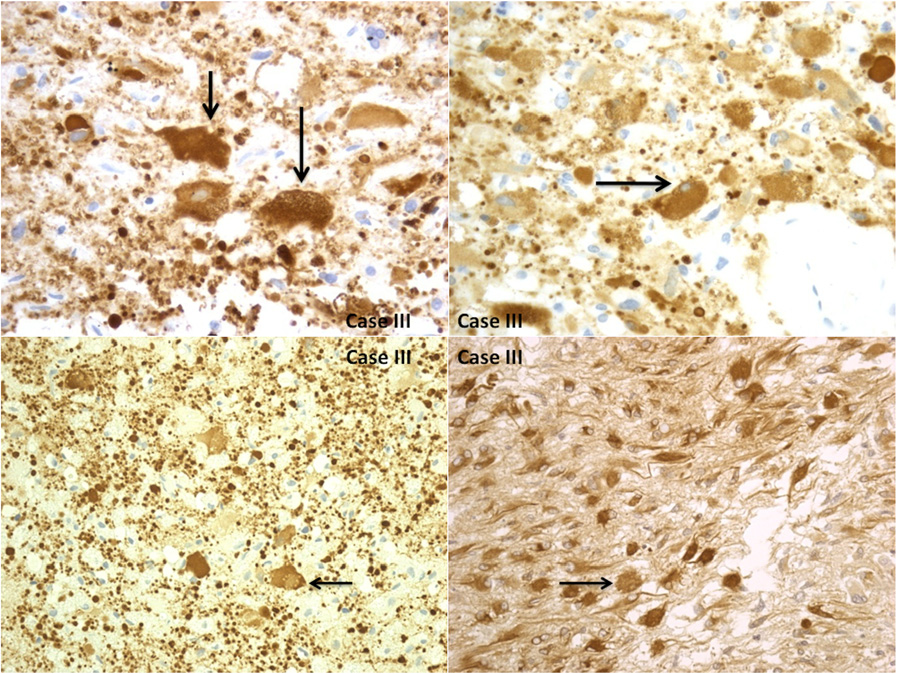
The cells have large nucleus and a prominent nucleolus, have a certain resemblance to the neurons, but are GFAP +.

**Figure 7:**
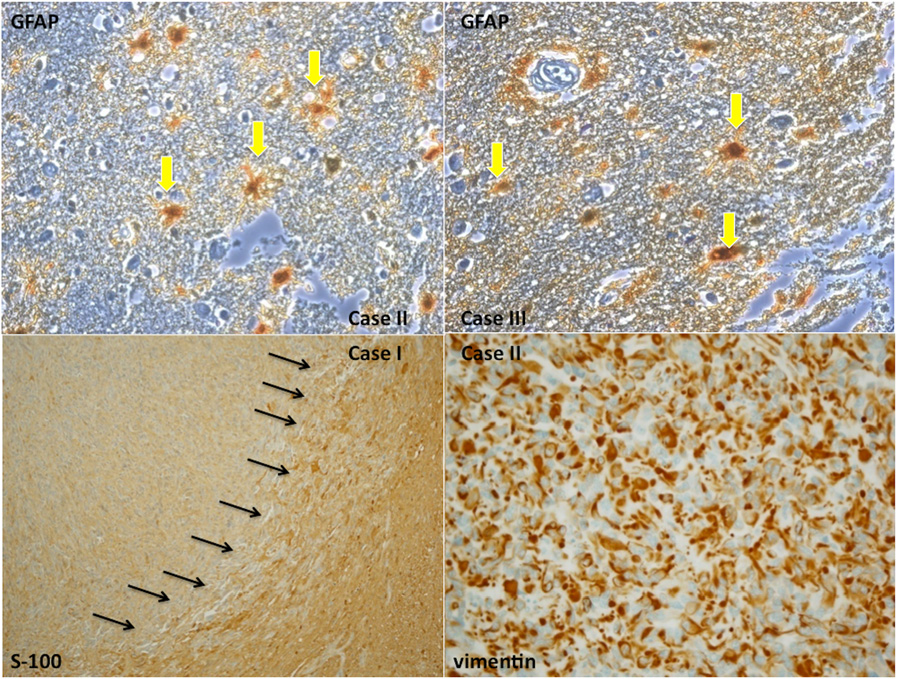
**Trapped reactive astrocytes were large, stellate, often peri-vascular and intensely reactive for GFAP.** The tumor cells were weakly immunoreactive with vimentin (case 2) and S-100 (case 1).

The immunohistochemical examination of the brain specimens revealed a positive reaction for antibodies anti-GFAP (glial fibrillary acidic protein) (Figures 5, 6), CD68, vimentin and S-100 (Figure 7). Reactions for NSE (neuron-specific enolase), smooth muscle actin**,** CD34, cytokeratins MNF (monoclonal neurofilarnent) 116, EMA (epithelial membrane antigen), synaptophysin, HMB45 (Human Melanoma Black) were negative. The other organs showed signs of central dysregulation (pulmonary oedema).

#### Western blot analysis

**Figure 8:**
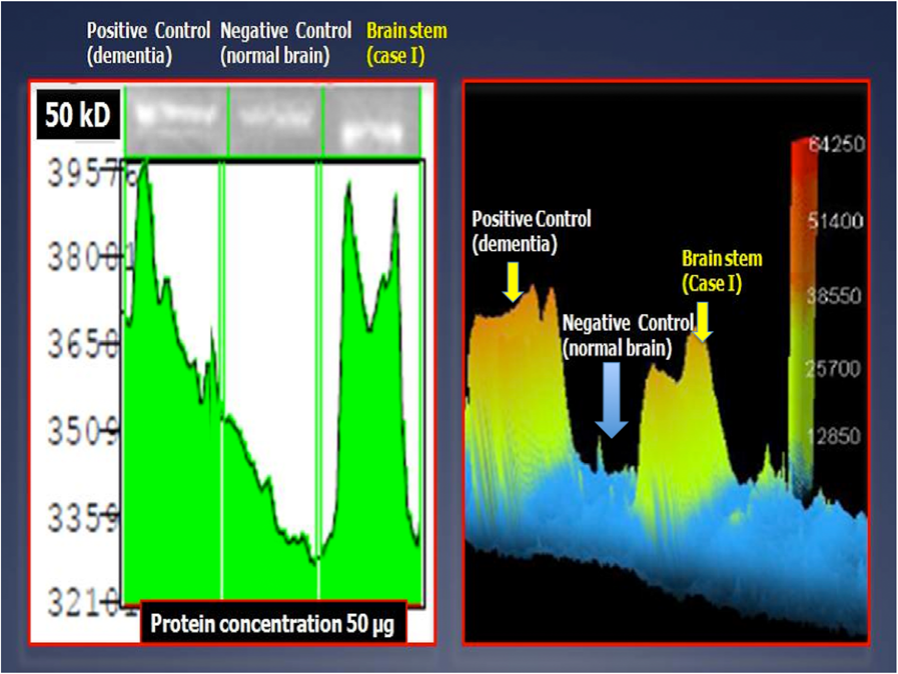
**Western blot.** Proteomic analysis shows the different bands and different concentrations of GFAP in brain stem of case 1, positive control (dementia cases) and negative control (normal brain).

Proteomic analysis was performed using Western blot. The positive reaction for GFAP was confirmed by Western blotting demonstrating different bands and different concentrations of GFAP in brain tumoural sites, compared with positive (dementia cases) and negative control (Figure 8).

The exitus was attributed in the second and third case to brain oedema and massive haemorrhage into the glioblastoma from erosion of vessels, with an increase in intracranial pressure and compression of cerebrospinal fluid circulation, whereas in the first case death can be explained by distortion and compression of the medulla by the tumour with consequent acute central dysregulation due to glioblastoma corresponding to WHO grade IV.

## Conclusions

Glioblastoma is highly malignant astrocytic glioma that appears to arise either de novo or in transition from diffuse astrocytoma and anaplastic astrocytoma. Glioblastomas that arise in transition from an often sizable, better-differentiated astrocytic tumor have been referred to as secondary glioblastomas [26]. Other glioblastomas, primary glioblastomas, are densely cellular and homogeneously anaplastic, and exhibit none of the less cellular and better-differentiated components seen in secondary tumors. It remains a matter of debate whether these “primary” variants are truly malignant de novo or have overrun and obscured a precursor lesion.

A med-line literature research was performed for the period of 1993 through 2012, using the thesaurus’ terms “sudden death” and “glioblastoma” in combination (see Table 1) [1,20,25,27-29]. In the current literature are published only 11 cases of sudden death due to glioblastoma multiforme (10 men and 1 woman), age (range) 10 weeks-75 years. In 7 cases there were no neurological symptoms before the death and the patients were found suddenly death [1,20,25]. In one case there was headache 2 hours before death [30], in 1 case the man was disoriented, slow, somnolent, five days before the death [28] and in a case of the child of 10 weeks there was irritability for one day and large vomit before the death (Table 1) [29].

**Table 1:** Cases of sudden death due to glioblastoma multiforme published in the current literature

	**Authors**	**Sex**	**Age (years)**	**Topography**	**Size of tumor (cm)**	**Symptom**
1	Sutton JT et al. (2010)	M	7	Right frontal lobe	7	2 hours before death complying of a headache
2	Vougiouklakis T. et al. (2006)	M	34	Third ventricle, at level of the foramen of Monro	6,9	No neurological symptoms. The man was found unconscious in bed
3	Shiferaw K. et al. (2006)	M	48	Right frontal lobe	4	Patient with schizophrenia. Five day prior death, the man was disoriented, slow, and somnolent.
4	Elgamal EA. et al. (2006)	M	10 week	Left parietal lobe	/	Irritability excessively for 1 day and large vomit
5	Matschke J. et al. (2005)	F	33	Right fronto-parietal lobe	/	No neurological symptoms. The woman was found dead in her apartment
6		M	52	Left cingulated gyrus with infiltration of the thalamus	/	No neurological symptoms. The man suddenly collapsed at home
7		M	75	Left cerebellar hemisphere with infiltration of adjacent brainstem structures	/	No neurological symptoms. The man was found lying dead in his bed
8	Eberhart C.G. et al. (2001)	M	39	left frontal lobe	5	No neurological symptoms. The man was found unresponsive on the bathroom
9		M	35	Right cerebral hemisphere showed a large mass lesion, involving the basal ganglia and internal capsule	5	No neurological symptoms. The man was found unresponsive
10		M	45	Right frontal lobe	4	The man died after his automobile left the road at a high rate of speed and impacted a tree.
11	Matsumoto H. (1993)	M	20	Left temporal lobe	/	/
12	Present paper	M	71	Right medulla and right temporal lobe	2	Headache, confusional state and difficulty in walking few hours before the death
13		M	79	Left temporal lobe	3	Confusion, slackening, sleepiness, and tremor of the upper limbs start few days before the death
14		M	43	Left frontal lobe	3,5	No neurological symptoms. The man was found dead in a slope near the track of the railway.

In the presented cases, the third one showed no neurological symptoms before the death and in the others cases there was a mild neurological symptomatology few hours before the death.

In recent years the concept of two distinct glioblastoma subtypes has been developed, combining clinical, morphological and genetic data. From this concept has emerged a clinical/molecular distinction of “primary” and “secondary” glioblastomas, although it is unclear how to distinguish this differentiation, or the extent to which it is therapeutically and prognostically relevant [31-33]. On the whole secondary types present in younger patients, more often women who have a longer duration of symptoms, and usually lie in the cerebral hemispheres. These tumors have a high frequency of mutation of p53 tumor suppressor gene on chromosome 17p with accumulation of p53 protein, but infrequent amplification of epidermal growth factor receptor (EGFR) involved in control of cell proliferation [32-35]. Loss of chromosome 19q in the region of a presumed tumor suppressor gene(s) as yet unidentified is more common in secondary tumors [36].

**Figure 9:**
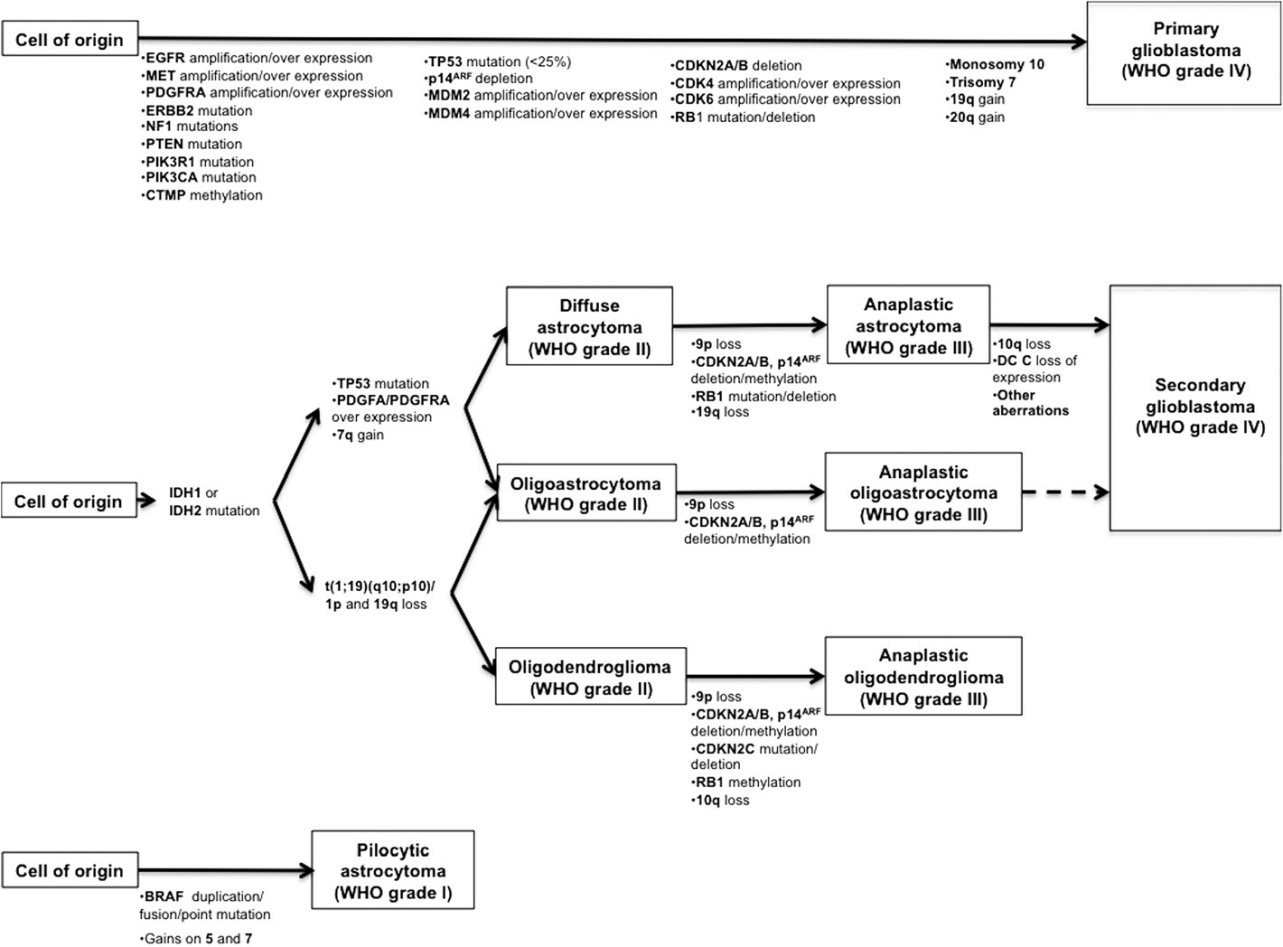
**Primary and secondary glioblastomas: distinct genetic pathways.** (Modified from 39).

The considerably more frequent primary form of glioblastoma appears more abruptly and generally later in life than the secondary type, and more often in men. Genetically, this variant less often has mutations of p53, but exhibits complex genetic abnormalities including deletions, often homozygous, of p16. Amplification of EGFR occurs in 30 to 40 percent of such lesions, and is commonly associated with homozygous deletions in p16^INK4a^[32,33]. Most of molecular and genetic markers are still lacking confirmation; currently, only two markers, O^6^-methylguanine-DNA methyltransferase (MGMT) promoter methylation and isocitrate dehydrogenase1 (IDH1) mutations, are commonly accepted genetic biomarkers for patients with glioblastoma [37]. As it has been authoritatively stated, primary and secondary glioblastomas are distinct disease entities and develop through distinct genetic pathways with different mRNA and protein expression profiles. Most of these genetic alterations can be ascribed to a determined set of functional pathways (Figure 9). These differences may affect sensitivity to radio- and chemotherapy and should thus be considered in the prognosis and response to therapy, but the genetic analysis is not so relevant for forensic purposes being fundamental, however, for this aim, the completion of diagnostic morphological investigations [38,39].

In the cases of our observation, the clinical and morphological data are in agreement to the diagnosis of primary glioblastoma. In all cases, in fact, the tumor was very aggressive and there weren't clinical symptoms before the death. Like astrocytomas of lower grade, glioblastomas may be discovered on evaluation for seizures or headache but unlike lower-grade lesions whose infiltrating and insinuating qualities carry the cells unobtrusively into intact parenchyma with little resultant mass effect, at least initially, glioblastoma are often expansive and edema generating. As a result, they are more likely to produce frank neurological deficits and sign of increased intracranial pressure: a subset present in sudden, stroke-like fashion as a consequence of intratumoral hemorrhage.

The features of intratumoral vascular proliferations, in fact, have a constant correlation with the prognosis. In glioblastoma vascular proliferation assumes two forms. Most common is a well-known variant that forms globular masses resembling the glomerular tufts of the kidney, this proliferation, now referred to as “microvascular proliferation”. The second form of vascular hyperplasia has a more legitimate claim to the term “endothelial proliferation” since it is intraluminal and consists largely of endothelial cells within small to medium-sized vessels. Endothelial proliferation is less common than glomeruloid microvascular proliferation and it appears to have a more constant correlation with high-grade gliomas and a poor prognosis.

When evaluating cases of sudden death due to undiagnosed glioblastoma there were several problems in defining the most probable causes of death. Although modern diagnostic imaging techniques have revolutionized the diagnosis of brain tumors, the autopsy and the careful gross examination and section of the fixed brain (with coronal section) is still the final word in determining exact location, topography, mass effects and histology and secondary damage of brain tumor and contributed the elucidation of the cause of death [40]. Immunohistochemistry and proteomic analysis are mandatory in such cases [41-43].

## Consent

Written informed consent was obtained from the patient's relatives for publication of this case report and any accompanying images.

## Competing interests

The authors declare that they have no competing interests.

## Authors' contributions

IR and VF equally contributed to this article and conceived the study. RZ and ET wrote the manuscript. MN, FDS, RP, CP and FV made the pathological explorations. All authors read and approved the final manuscript.
